# Treatment for Covid-19 with SARS-CoV-2 neutralizing antibody BRII-196(Ambavirumab) plus BRII-198(Lomisivir): a retrospective cohort study

**DOI:** 10.1186/s40360-024-00753-7

**Published:** 2024-04-19

**Authors:** Qin Yalan, Hao Lingfang, Liu Xisong, Liang Run, Zhang Junjing, Zhang’ An

**Affiliations:** 1 Department of Critical Care Medicine, The Second Affiliated Hospital of Chongqing Medical University, 76# Linjiang Road, Yuzhong District, 400016 Chongqing, China; 2 Department of Oncology, The Hohhot First Hospital, 010030 Hohhot, China; 3 Department of Critical Care Medicine, Chongqing Public Health Treatment Center, 400030 Chongqing, China; 4 Department of Hepatobiliary Surgery, The Hohhot First Hospital, 150# South Second Ring Road, Yuquan District, Inner Mongolia Autonomous Region, 010030 Hohhot, China

**Keywords:** COVID-19, BRII-196, BRII-198, Neutralizing antibody therapy, Therapeutic effect

## Abstract

**Background:**

Monoclonal antibody therapy for Covid-19 springs up all over the world and get some efficiency. This research aims to explore the treating effect of BRII-196(Ambavirumab) plus BRII-198(Lomisivir) on Covid-19.

**Methods:**

In this retrospective cohort research, patients received standard care or plus BRII-196 /BRII-198 monoclonal antibodies. General comparison of clinical indexes and prognosis between Antibody Group and Control Group was made. Further, according to the antibody using time and patients’ condition, subgroups included Early antibody group, Late antibody group, Mild Antibody Group, Mild Control Group, Severe Antibody Group and Severe Control Group.

**Results:**

Length of stay(LOS) and interval of Covid-19 nucleic acid from positive to negative of Antibody Group were 12.0(IQR 9.0–15.0) and 14.0(IQR 10.0–16.0) days, less than those(13.0 (IQR 11.0–18.0) and 15.0 (IQR 12.8–17.0) days) of Control Group(*p* = 0.004, *p* = 0.004). LOS(median 10days) of Early Antibody Group was the shortest, significantly shorter than that of Control Group (median 13days)(*p* < 0.001). Interval(median 12days) of Covid-19 nucleic acid from positive to negative of Early Antibody Group also was significantly shorter than that of Control Group(median 15days) and Late Antibody Group(median 14days)(*p* = 0.001, *p* = 0.042). LOS(median 12days) and interval(median 13days) of Covid-19 nucleic acid from positive to negative of Mild Antibody Group was shorter than that of Mild Control Group(median 13days; median 14.5days)(*p* = 0.018, *p* = 0.033).

**Conclusion:**

The neutralizing antibody therapy, BRII-196 plus BRII-198 could shorten LOS and interval of Covid-19 nucleic acid from positive to negative. However, it didn’t show efficacy for improving clinical outcomes among severe or critical cases.

**Supplementary Information:**

The online version contains supplementary material available at 10.1186/s40360-024-00753-7.

## Introduction

Covid-19 pandemic have been accompanying us for about 3 years so far. Different from Severe Acute Respiratory Syndrome, Covid-19 caused slight symptoms like fever, cough, fatigue and muscle ache at the very beginning and patients prone to be self-cured [1]. However, with time going on, about 20% infects turned into critical type accompanying acute respiratory distress syndrome(ARDS) and achieved a bad prognosis [2]. And as the virus mutating, like the current circulating strain, Omicron, the infectiousness gets stronger [3].

Reducing Covid-19 infection rates and improving cure rates are of great importance. Including corticosteroids, traditional Chinese medicine, convalescent plasma and so on, systemic treatment options are quite limited in terms of effectiveness and safety [4–6]. At first, active immunization through vaccines was thought to terminate the disaster. However, as the virus strain mutating, the effectiveness of vaccine is limited. Following convalescent plasma therapy and passive immunotherapy progressing, novel anti-virus drugs like small molecules and neutralizing antibodies are emerging at the historic moment.

As we know, interaction of the receptor-binding domain (RBD) of spike S protein of the severe acute respiratory syndrome coronavirus 2(SARS-CoV-2) with the host epithelial angiotensin-converting enzyme 2 (ACE2) is the leading event for the viral entry [7–8]. After gaining entry into the cytoplasm, SARS-CoV-2 utilizes the JAK-STAT pathway to target the lymphocytes, leading to symptoms including fever, cough, fatigue, throat pain and so on [9]. Neutralizing monoclonal antibodies could target the spike (S) glycoproteins on the SARS-CoV-2 surface that mediate entry into host cells and prevent virus entry [10]. BRII-196 and BRII-198, is one kind of neutralizing antibody developed by Tsinghua University, and the 3rd People’s Hospital of Shenzhen. On July 7,2022, as the first approved neutralizing antibody combination treatment drug with proprietary intellectual property right in our country, it was introduced in public. The second Accelerating COVID-19 Therapeutic Interventions and Vaccines platform(ACTIV-2) has verified its effectiveness and safety in adults with mild or normal type COVID-19 [11].

However there is no clinical trial to get the same conclusion and apply to severe or critical cases. Our research enrolled patients to explore the effectiveness of BRII-196 and BRII-198 in both mild and severe patients.

## Methods

### Research design and participants

This retrospective cohort study enrolled 340 COVID-19 patients in total admitting to the Hohhot First Hospital and Chongqing Public Health Treatment Center confirmed by nucleic acid tests from October to November, 2022.

Our research was in three parts. First, to explore the effectiveness of monoclonal antibodies, we divided patients no matter which type into Antibody Group and Control Group, according to the therapies including BRII-196 and BRII-198 or not. Clinical indexes and prognosis were compared between the two groups.

Second, to study whether the antibody using time affects patients’ clinical manifestation and prognosis, we divided the Antibody Group into two parts depending on the interval of admitting and using antibody. Since the interval equal or less than 5 days, this part patients called Early Antibody Group and the rest called Late Antibody Group. Clinical indexes and prognosis were compared between the two groups and Control Group.

**Fig. 1:**
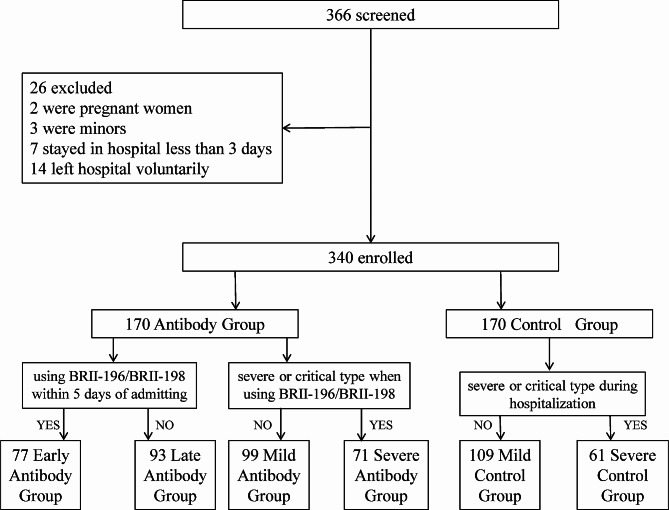
Enrollment and classification

Third, to explore whether there are different effects on mild and severe cases, we divided both Antibody Group and Control Group into mild and severe parts, named Mild Antibody Group, Severe Antibody Group, Mild Control Group and Severe Control Group separately. For Antibody Group, the criteria of dividing was the clinical type when using antibody therapy while for control group the criteria was the worst clinical type during hospitalization. Clinical indexes and prognosis were compared between Mild groups and Severe groups respectively.(Figure 1).

### Inclusion criteria

1. Over 18 years old.
2. Primary onset of COVID-19 and meet the diagnostic criteria of COVID-19 of < New coronavirus pneumonia prevention and control program(9th ed.)> [12].
3. Patients went through the whole regular treatment and got a definite outcome(discharged or dead).

### Exclusion criteria

1. Length of stay(LOS) less than 3 days.
2. Treatment interrupting or unplanned discharging for patients’ reason.
3. Less than 18 years old.
4. Pregnant women.

### Data collection

Epidemiological, demographic, clinical, laboratory, treatment, and outcome data were extracted from electronic medical records using a standardized data collection form by several experienced clinical physicians. All data were checked by at least two workers (Qin Yalan, Liu Xisong and Hao Lingfang). If there exited any doubt, we asked the doctor in charge for details to complete these data.

### Laboratory tests

We collected the data including indexes of routine blood examinations, interleukin-6 (IL-6), procalcitonin(PCT), D-dimer, prothrombin time(PT), activated partial thromboplastin time(APTT), total bilirubin(TBIL), creatinine(Cr), blood urea nitrogen(BUN), C-reactive protein(CRP), troponin I(TNI), N-terminal pro-brain natriuretic peptide(NT-proBNP) and Severe Acute Respiratory Syndrome coronavirus 2 Reverse Transcription-Polymerase Chain Reaction Cycle Threshold (SARS-CoV-2 RT-PCR CT) values during hospitalization on the admitting day(Day1), the seventh day(Day7) and the fourteenth day(Day14) separately.

### Definition and states

All the patients enrolled were diagnosed according to the diagnostic criteria of < New coronavirus pneumonia prevention and control program(9th ed.)> [12] as below: The mild type had slight symptoms without manifestation in radiograph while the normal type accompanying image change. Severe type ought to match to at least one of the three points(respiratory distress, Respiratory Rate ≥ 30 times/min; At rest, oxygen saturation ≤ 93%; Oxygen Index(OI) ≤ 300mmHg) and critical type also in accord with at least one of the three points (Respiratory failure requiring mechanical ventilation; Go into shock; Combined with other organ failure requiring intensive care). In this study, we defined the mild and normal type as Mild, and the severe and critical type as Severe.

In order to ensure the homogenization of patient management, discharged patients should meet the criteria as absence of fever for at least 3 days, substantial improvement in both lungs in chest CT, clinical remission of respiratory symptoms, and two successive throat-swab samples negative for SARS-CoV-2 RNA obtained at least 24 h apart. Otherwise the case should be excluded.

### Treatment

General treatment included oxygen support, nutrient support and complication treatment. 5 mg azvudine once a day or 400 mg paxlovid twice a day for successive five days were used against virus while immunopotentiator including thymalfasin and thymopentin were used to improve immunity for partial patients. And some patients received antibiotics, glucocorticoid or immunoglobulin treatment.

### Statistical analysis

All analyses were conducted with SPSS 26.0. Continuous variables were expressed as medians with interquartile ranges for the variables were not normal distribution. Categorical variables were summarized as the counts and percentages. The Mann-Whitney U test or Kruskal-Wallis H test was applied to continuous variables, and the Fisher exact test or Pearson χ2 test was used for categorical variables. In pairwise comparison of independent samples, *p* value was adjusted by Bonferroni correction method. Differences were statistically significant at *p* < 0.05.

## Results

At the beginning, data of 366 patients were retrieved from the hospital information system. However, 14 left hospital voluntarily; 2 were pregnant women; 3 were minors and 7 stayed in hospital less than 3 days. At last, there were 340 patients enrolled into the research in total, as Antibody Group and Control Group each involving 170. Their median age were 72.0(IQR 58.0–81.0) years old. Among them, the median age of Antibody Group and Control Group were 75.0 (IQR 63.8–83.3) and 70.0 (IQR 53.8–78.0) years old severally(72.0 vs. 75.0, *p*<0.001). Although the Antibody Group were older than the other, two groups had almost approximate gender proportion. There were 111 males in Antibody Group while 95 in Control Group. They almost had comorbidity, such as chronic obstructive pulmonary disease(COPD), lung cancer, coronary heart disease(CHD), hypertension, diabetes mellitus(DM) and chronic kidney disease(CKD). The incidence were 24.7%(42/170), 2.9%(5/170), 23.5%(40/170), 43.5(74/170), 20.6%(35/170) and 8.2%(14/170) in Antibody Group comparing 18.2%(31/170), 1.8%(3/170), 17.6%(30/170), 37.6%(64/170), 22.9%(39/170) and 2.9%(5/170) in Control Group. And there was no statistical difference between the two groups. In total, there were 69(20.3%) classified Severe type when admitting. For treatment strategies, 63.5% accepted Azvudine or Paxlovid against SARS-cov-2. Only 5.6% patients used immunoglobulins. According to statistics, there were 17.6% patients using cefuroxime, 63.2% patients using piperacillin tazobactam sodium, 7.6% patients using meropenem, and the rest using cefoperazone sodium and sulbactam sodium in total. 57.6% patients used glucocorticoid for 3–5 days. There was no significant difference in treatment between two groups(Table 1).

**Table 1:** Baseline demographic, disease, and clinical characteristics

Variable	General (*N* = 340)	Antibody Group(*N* = 170)	Control Group (*N* = 170)	*P* value
Age(years old)	72.0(58.0, 81.0)	75.0(63.8, 83.3)	70.0(53.8, 78.0)	<0.001
Sex, male(n,%)	206, 60.6	111, 65.3	95, 55.9	0.076
Severe type rate(n,%)	69, 20.3	40, 23.5	29, 17.1	0.138
Comorbidity(n,%)				
COPD	73, 21.5	42, 24.7	31, 18.2	0.146
Lung cancer	8, 2.4	5, 2.9	3, 1.8	0.723
CHD	70, 20.6	40, 23.5	30, 17.6	0.180
Hypertension	138, 40.6	74, 43.5	64, 37.6	0.269
DM	74, 21.8	35, 20.6	39, 22.9	0.599
CKD	19, 5.6	14, 8.2	5, 2.9	0.034
None	50, 14.7	19, 11.2	31, 18.2	0.066
Using immunoglobulins(n,%)	20, 5.9	9, 5.3	11, 6.5	0.645
Using Azvudine or Paxlovid (n,%)	216, 63.5	115, 67.6	101, 59.4	0.115

Note: COPD: chronic obstructive pulmonary disease; CHD: coronary heart disease; DM: diabetes mellitus; CKD: chronic kidney disease

**Fig. 2:**
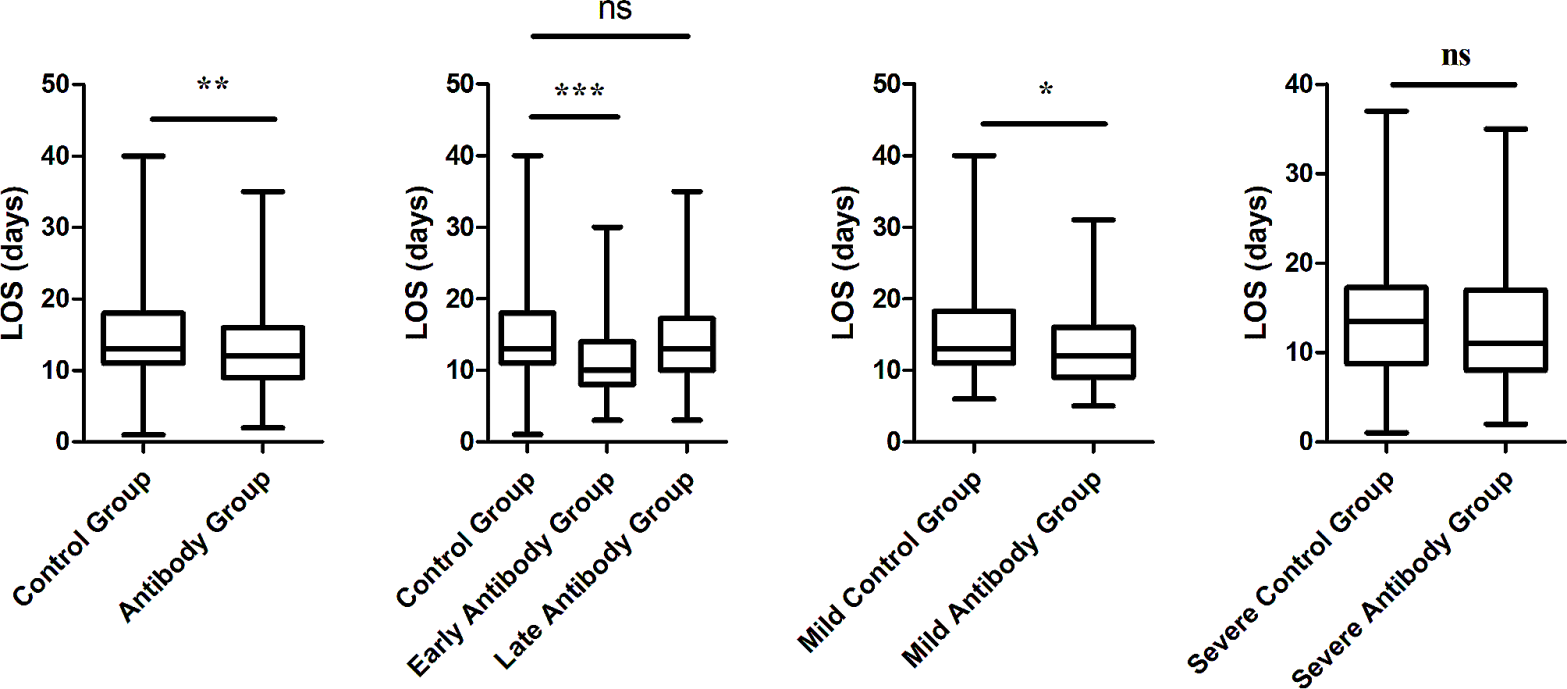
Comparison of length of stay between groups Note: “*” means *p* < 0.05, “**” means *p* < 0.01, “***” means *P* < 0.001, “ns” means not significant

**Fig. 3:**
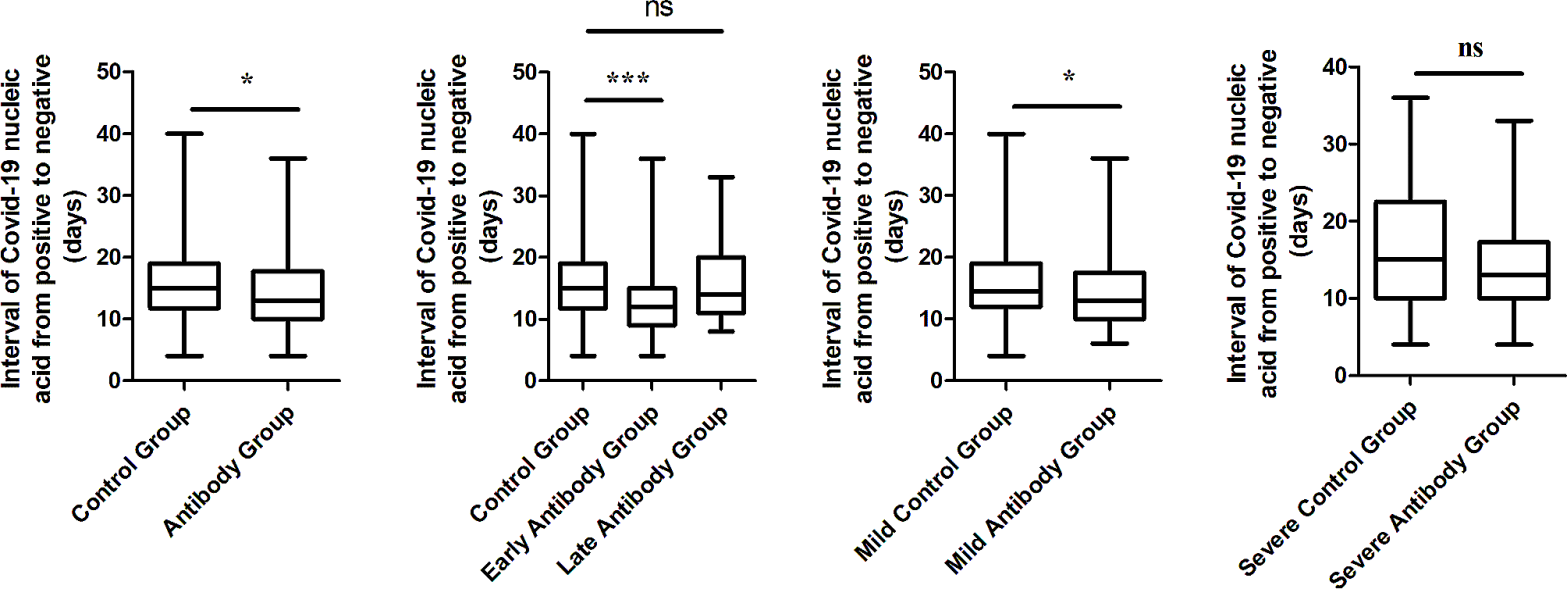
Comparison of interval of Covid-19 nucleic acid from positive to negative between groups Note: “*” means *p* < 0.05, “**” means *p* < 0.01, “***” means *P* < 0.001, “ns” means not significant

In the first part, we compare the clinical status and outcome between Antibody Group and Control Group. On admitting day(Day 1), The lymphocyte level of Antibody Group was 0.75(IQR 0.46–1.12)*10^9 /L, lower than Control Group 0.85(IQR 0.54–1.37)*10^9 /L while IL-6, D-dimer, Cr, BUN, CRP and NT-proBNP of Antibody Group were 68.0(IQR 16.1-138.8), 0.91(IQR 0.58–2.20), 81.4(IQR 62.5-108.5), 6.16(IQR 4.69–9.31), 22.2(IQR 9.6–71.5) and 617.7(IQR 146.8-4067.7) separately, higher than the other group, respectively being 0.55(IQR 0.26–1.25), 11.8(IQR 11.3–12.8), 71.1(IQR 56.7–88.2), 5.23(IQR 4.26–7.41), 11.9(IQR 3.7–39.9), 279.5(IQR 64.7–1309.0)(*P* < 0.05). They had similar level in SARS-CoV-2 RT-PCR CT value, WBC, Neutrophil, PCT, APTT and TBIL. There exists significant difference in PT and TNI between the two groups. However, those indexes are just slightly elevated, and the coagulation and heart function were often normal. On Day 7, the lymphocyte level of Antibody Group was 0.75(IQR 0.50–1.09)*10^9 /L, still lower than Control Group 1.02(IQR 0.53–1.49)*10^9 /L. At the same time, WBC level of Antibody Group was also lower than the other(4.66(IQR 3.49–7.16) vs 5.69(IQR 4.04–7.98)). Similaryly, IL-6, Cr, BUN, CRP and NT-proBNP of Antibody Group were 51.5 (IQR 22.8-144.3), 75.6 (IQR 59.4-133.9), 7.62 (IQR 4.26–13.32), 48.3 (IQR 14.4–92.5) and 2052.3 (IQR 480.0-4425.0) separately, higher than the other group, respectively being 21.8(IQR 8.8–61.3), 65.4(IQR 51.5–86.0), 5.57(IQR 3.89–8.89), 27.8(IQR 6.0-66.2), 547.5(IQR 203.3-2222.1). On Day 14, the lymphocyte of two groups were 0.82 (IQR 0.43–1.19) and 1.02 (IQR 0.55–1.44) and there was no statistic difference. Cr, BUN, CRP and NT-proBNP level of Antibody Group still slightly higher than Control Group. Nevertheless, LOS and interval of Covid-19 nucleic acid from positive to negative of Antibody Group were 12.0 (IQR 9.0–15.0) and 14.0 (IQR 10.0–16.0)days, less than those(13.0 (IQR 11.0–18.0)and 15.0 (IQR 12.8–17.0)days) of Control Group(12.0 vs. 13.0, *p* = 0.004; 14.0 vs. 15.0, *p* = 0.004). Finally, in Antibody Group, 42(24.7%) transferred to intensive care unit(ICU), 41(24.1%) mild patients converted to severe, and 12(7.1%) died. In Control Group, 39(22.9%) transferred to intensive care unit, 36(21.2%) mild patients converted to severe, and 7(4.1%) died. The difference between them was not significant(24.7% vs. 22.9%, *p* = 0.703; 24.1% vs. 21.2%, *p* = 0.517, 7.1% vs. 4.1%, *p* = 0.238).(Supplemental Table 1, Figs. 2 and 3).

The basic condition of two groups were not totally comparable due to the retrospective study. The Antibody Group seemed older, worse in renal function and rather intense of inflammatory response in the initial state.

In the second part, Early Antibody Group, Late Antibody Group and Control Group were compared to each other. There were 77 in Early Antibody Group while there were 93 in Late Antibody Group.

LOS(10.0(IQR 8.0–14.0)) of Early Antibody Group was the shortest, significantly shorter than that of the Control Group(13.0(IQR 11.0–18.0))(10.0 vs. 13.0, *p* < 0.001). Similarly, interval(13.0(IQR 9.0–15.0)) of Covid-19 nucleic acid from positive to negative of Early Antibody Group also was significantly shorter than that of Control Group(15.0(IQR 12.8–17.0)) and Late Antibody Group(15.0(IQR 11.0-18.5))(13.0 vs. 15.0, *p* = 0.001; 13.0 vs. 15.0, *p* = 0.042). However, there was no marked difference between each group in fatality, rate of condition aggravation and ICU attending rate.(Figures 2 and 3).

As for biomarkers, three groups had parallel level in viral RT-PCR CT values, WBC, Neutrophil, lymphocyte, TBIL and TNI from admitting to discharging. PCT, IL-6, D-dimer, CRP and NT-proBNP of Early Antibody Group were higher than that of Control Group when admitting. At Day14, Early Antibody Group and Control Group had similar level in above biomarkers(*P* > 0.05). Most of these biomarkers were at similar level in Early Antibody Group and Late Antibody Group from admitting to Day14. And also, there was no significant difference between Late Antibody Group and Control Group in most biomarkers. During two week treatment, inflammatory indexes of Early Antibody Group and Control Group manifested decreasing trends while those of Late Antibody Group presented an increasing trend before decreasing.(Supplemental Table 2).

In the last part, Mild Antibody Group was compared with Mild Control Group and Severe Antibody Group was compared with Severe Control Group. There were 99 in Mild Antibody Group, 71 in Severe Antibody Group, 109 in Mild Control Group, and 61 in Severe Control Group, separately.

LOS(12.0(IQR 9.0–16.0)days) and interval(13.0(IQR 10.0–18.0)days) of Covid-19 nucleic acid from positive to negative of Mild Antibody Group was shorter than that of Mild Control Group(13.0(IQR 11.0-18.3)days); 14.5(IQR 12.0–19.0)days)(12.0 vs. 13.0,*p* = 0.018; 13.0 vs. 14.5, *p* = 0.033). But they had similar fatality. Talking about SARS-CoV-2 RT-PCR CT value, Mild Antibody Group(median 25.73 and 25.35) were higher than Mild Control Group(median 23.25 and 24.13) at the beginning, becoming approximate after one week. And the lymphocyte count of Mild Antibody Group was lower than that of Mild Control Group on Day 1 while they came to the similar level at last. Except Cr, D-dimer, PT and APTT, the other indexes of them were almost approximate from admitting to Day 14. Although PT and APTT were different between two group, they were both in normal range. Cr of Mild Antibody Group was 78.9(IQR 59.4–95.9), higher than that of Mild Control Group(67.2(IQR 56.8–81.7)) at the beginning. However, it decreased 66.5(IQR 54.5–108.0)at Day 14, similar with the value of Mild Control Group(61.2(IQR 49.0-78.3)).(Supplemental Table 3, Figs. 2 and 3).

Disparately, LOS(median 11days) and interval(median 13days) of Covid-19 nucleic acid from positive to negative of Severe Antibody Group was not shorter than that of Severe Control Group(median 13.5days; median 15days)(*p* > 0.05). The viral RT-PCR CT value and biomarker level of two groups were comparable when admitting. However, on Day 7, neutrophil and lymphocyte count were lower in Severe Antibody Group while PCT, IL-6 were higher. At last, Cr and NT-proBNP were higher in Severe Antibody Group.(Supplemental Table 4, Figs. 2 and 3).

## Discussion

In this research, we found that BRII-196 plus BRII-198 as one kind of neutralizing antibody therapy, could shorten LOS and interval of Covid-19 nucleic acid from positive to negative. And it might also reduce inflammatory response for Covid-19 patients.

In general comparison of Antibody Group and Control Group, BRII-196 plus BRII-198 showed the potential to inhibit SARS-CoV-2 replication and cut down LOS and the interval of Covid-19 nucleic acid from positive to negative. Also, it performed the ability to reduce inflammatory reaction conducting inflammatory indexes( IL-6 and CRP) decreased in Antibody Group, although above indexes were not significantly lower than the Control Group at the end. Considering worse initial state, inflammatory biomarkers and Cr of Antibody Group decreased to the similar level of Control Group in the end, which could demonstrate anti-inflammatory effect of BRII-196 plus BRII-198 as well. As reported, SARS-CoV-2 enters into a human cell through its receptor-binding domain(RBD) on spike (S) protein binding to ACE2, invoking a hyperinflammatory state driven by multiple cells and mediators like interleukin IL-1, IL-6, granulocyte-macrophage colony-stimulating factor, complement and so on [13]. Early research have reported BRII-196, recognizes and binds an epitope overlapping with the ACE2-binding site on the spike protein of SARS-CoV-2. BRII-198 binds to a different epitope on the spike protein with synergistic effect when combined with BRII-196. Moreover, in vitro assay, it was found that escape mutants were not generated following treatment with a cocktail composed of non-competing antibodies [14]. In Jin Yong Kim’s study, the CT-P59, another potent neutralizing antibody against various SARS-CoV-2 isolates, identified through screening human monoclonal antibodies from the peripheral blood mononuclear cells of a SARS-CoV-2 convalescent patient, has been demonstrated the potential antiviral and clinical efficacy in patients with mild SARS-CoV-2 infection [15].

Similar results occurring in the comparison of Early Antibody Group and Control Group, revealed the positive effect of monoclonal antibody therapy. ACTIV-2(NCTO4518410) has revealed similar evidence. It reported that patients who received Ambavirumab/Lomisivir therapy either within 5 days or 6-10days of symptom onset developed into hospitalization or dying less than placebo group [16]. However, in the subgroups analysis, it’s not found that the Late Antibody Group had better treatment effects than Control Group, even worse at the middle of hospitalization. Besides the reason of worse initial situation of Late Antibody Group, a randomised controlled trial of ACTIV-3 conducting by Dr Wesley H Self’s team indicated that neither sotrovimab nor BRII-196 plus BRII-198 showed efficacy for improving clinical outcomes among adults hospitalized with COVID-19 [17].

Jin Yong Kim found that CT-P59, a potent neutralizing monoclonal antibody against various SARS-CoV-2 isolates, had potential antiviral and clinical efficacy in patients with mild COVID-19. Ji-Min Seo found that CT-P63 was clinically safe and showed broad-spectrum neutralizing activity against SARS-CoV-2 variants in vitro and in vivo. LY-CoV555 (Eli Lilly/ AbCellera) and REGN-COV2 (Regeneron) also had been studied as medical antibodies against SARS-CoV2 [18–21]. There were few researches studying the BRII 196/BRII 198. In the third part, we found that the LOS and interval of Covid-19 nucleic acid from positive to negative of Mild Antibody Group were shorter than those of Mild Control Group. Coincidentally, in Anil Gupta’s study, it’s found that sotrovimab, formerly known as VIR-7831, an engineered human monoclonal antibody that neutralizes SARS-CoV-2 and multiple other sarbecoviruses, including SARS-CoV-1, reduced the risk of disease progression among high-risk patients with mild-to-moderate Covid-19 [22]. To seek more extensive indications for BRII-196 plus BRII-198 antibody therapy, we off-label used BRII-196 plus BRII-198 in severe and critical Covid-19 hospitalized patients. Nevertheless, BRII-196 plus BRII-198 antibody therapy didn’t show efficacy for improving clinical outcomes among adults hospitalized with severe or critical COVID-19.

Morbidity and mortality from COVID-19 remain substantial, creating an urgent need for more effective therapies for severely ill patients with COVID-19. We compared the Severe type between Antibody and Control Group, which is unprecedented to explore the effect of BRII-196 plus BRII-198 on severe and critical cases. Although, there was no significant difference in clinical improvement and prognosis between Severe Antibody Group and Severe Control Group.

There does exist some limitations in the study. First, it’s only including two medical center from north and south China separately, hard to represent all cases. And the sample size was relatively small. Second, there were two medical center, thus patients maybe got little different treatment during hospitalization, which could generate intervention bias. For example, the antibiotics, antiviral drug(azvudine or paxlovid), glucocorticoid and immunoglobulin using were not the same in each patient. Fortunately, there wasn’t difference between two groups in treating through statistical analysis. Third, as a retrospective cohort research, there existed data missing and omissions.

In conclusion, BRII-196 plus BRII-198 could reduce LOS and interval of Covid-19 nucleic acid from positive to negative and decrease inflammatory response in SARS-Cov-2 infection.

### Electronic supplementary material

Below is the link to the electronic supplementary material.

Supplementary Material 1

## Data Availability

The data that support the findings of this study are available from the corresponding author upon reasonable request.
