# Supplementary material for: Treatment for Covid-19 with SARS-CoV-2 neutralizing antibody BRII-196(Ambavirumab) plus BRII-198(Lomisivir): a retrospective cohort study
Source: BMC Pharmacol Toxicol. 2024 Apr 19;25:29. doi: 10.1186/s40360-024-00753-7 (PMC11027409; doi:10.1186/s40360-024-00753-7)
Supplement: Supplementary file 1 — Supplementary Material 1 [file 40360_2024_753_MOESM1_ESM.doc]

**SUPPLEMENTAL DIGITAL CONTENT**

Table of Contents :

Supplemental Table 1( Page 2-3)

Supplemental Table 2( Page 4-6)

Supplemental Table 3( Page 7-8)

Supplemental Table 4( Page 9-10)

**Supplemental Table 1 Biochemical indexes and prognosis comparison of Antibody Group and Control Group**

| **Indexes** | | **Antibody Group**  **(N=170)** | **Control Group**  **(N=170)** | **P value** |
| --- | --- | --- | --- | --- |
| **D1** | **SARS-CoV-2 RT-PCR CT values(O)** | 24.96(21.27,29.02) | 23.44(16.87,28.67) | 0.025 |
|  | **SARS-CoV-2 RT-PCR CT values(N)** | 24.79(20.79,28.78) | 24.42(19.03,28.93) | 0.380 |
|  | **WBC,*10^9/L** | 4.84(3.86,7.64) | 5.24(3.97,6.97) | 0.632 |
|  | **Neutrophil,*10^9/L** | 3.41(2.66,5.73) | 3.59(2.52,5.51) | 0.806 |
|  | **Lymphocyte,*10^9/L** | 0.75(0.46,1.12) | 0.85(0.54,1.37) | 0.022 |
|  | **PCT,ng/ml** | 0.141(0.080,0.523) | 0.104(0.059,0.220) | 0.017 |
|  | **IL-6,pg/ml** | 68.0(16.1,138.8) | 19.7(7.62,49.8) | 0.002 |
|  | **D-dimer,mg/l** | 0.91(0.58,2.20) | 0.55(0.26.1.25) | ＜0.001 |
|  | **PT,s** | 12.5(11.7,13.7) | 11.8(11.3,12.8) | ＜0.001 |
|  | **APTT,s** | 28.8(25.7,32.5) | 29.0(27.0,32.3) | 0.624 |
|  | **TBIL,umol/l** | 10.64(7.77,15.18) | 10.50(7.50,15.60) | 0.908 |
|  | **Cr,umol/l** | 81.4(62.5,108.5) | 71.1(56.7,88.2) | 0.002 |
|  | **BUN,mmol/l** | 6.16(4.69,9.31) | 5.23(4.26,7.41) | 0.023 |
|  | **CRP,mg/l** | 22.2(9.6,71.5) | 11.9(3.7,39.9) | 0.004 |
|  | **TNI,ng/ml** | 0.02(0.02,0.08) | 0.02(0.01,0.03) | 0.031 |
|  | **NT-proBNP,pg/ml** | 617.7(146.8,4067.7) | 279.5(64.7,1309.0) | 0.022 |
| **D7** | **SARS-CoV-2 RT-PCR CT values(O)** | 29.77（26.56，34.51） | 29.39（24.85，33.40） | 0.240 |
|  | **SARS-CoV-2 RT-PCR CT values(N)** | 29.91（26.12，33.98） | 30.29（25.24，34.30） | 0.860 |
|  | **WBC,*10^9/L** | 4.66（3.49，7.16） | 5.69(4.04,7.98) | 0.010 |
|  | **Neutrophil,*10^9/L** | 3.36（2.05，5.77） | 3.84(2.44,6.58) | 0.141 |
|  | **Lymphocyte,*10^9/L** | 0.75（0.50，1.09） | 1.02(0.53,1.49) | 0.004 |
|  | **PCT,ng/ml** | 0.300（0.113,1.310） | 0.120(0.061,0.480) | 0.018 |
|  | **IL-6,pg/ml** | 51.5（22.8，144.3） | 21.8(8.8,61.3) | 0.013 |
|  | **D-dimer,mg/l** | 1.30（0.50，4.04） | 0.77(0.53,1.93) | 0.077 |
|  | **PT,s** | 12.8（11.8，13.7） | 12.2(11.5,13.1) | 0.069 |
|  | **APTT,s** | 30.8（27.6，34.7） | 29.2(25.8,33.5) | 0.023 |
|  | **TBIL,umol/l** | 10.70（7.39，15.58） | 11.35(8.74,15.95) | 0.299 |
|  | **Cr,umol/l** | 75.6（59.4，133.9） | 65.4(51.5,86.0) | 0.005 |
|  | **BUN,mmol/l** | 7.62（4.26，13.32） | 5.57(3.89.8.89) | 0.014 |
|  | **CRP,mg/l** | 48.3（14.4，92.5） | 27.8(6.0,66.2) | 0.013 |
|  | **TNI,ng/ml** | 0.03（0.01，0.06） | 0.03(0.01,0.15) | 0.811 |
|  | **NT-proBNP,pg/ml** | 2052.3（480.0，4425.0） | 547.5(203.3,2222.1) | 0.004 |
| **D14** | **WBC,*10^9/L** | 6.14（4.66，8.93） | 6.03（4.42，8.32） | 0.539 |
|  | **Neutrophil,*10^9/L** | 4.65（3.09，7.51） | 3.80（2.78，5.68） | 0.262 |
|  | **Lymphocyte,*10^9/L** | 0.82（0.43，1.19） | 1.02（0.55，1.44） | 0.074 |
|  | **PCT,ng/ml** | 0.211（0.075，1.065） | 0.095（0.059，0.396） | 0.066 |
|  | **IL-6,pg/ml** | 24.4（13.0，106.8） | 20.7（7.3，49.8） | 0.165 |
|  | **D-dimer,mg/l** | 1.11（0.56，3.89） | 1.23（0.50，4.23） | 0.939 |
|  | **PT,s** | 13.2（12.2，14.1） | 12.3（11.8，13.3） | 0.038 |
|  | **APTT,s** | 28.7（26.8，32.5） | 26.8（24.7，30.5） | 0.054 |
|  | **TBIL,umol/l** | 11.39（8.41，18.05） | 12.43（6.91，15.30） | 0.401 |
|  | **Cr,umol/l** | 75.8（59.8，119.9） | 62.5（45.8，78.4） | 0.002 |
|  | **BUN,mmol/l** | 9.49（4.60，15.66） | 6.18（4.21，9.06） | 0.046 |
|  | **CRP,mg/l** | 46.2（12.4，106.8） | 22.6（2.7，62.5） | 0.012 |
|  | **TNI,ng/ml** | 0.02（0.01，0.06） | 0.03（0.01，0.05） | 0.935 |
|  | **NT-proBNP,pg/ml** | 1569.1（783.2，4172.1） | 562.4（277.1，1649.9） | 0.023 |
| **ICU attending rate（n,%）** | | 42，24.7 | 39，22.9 | 0.703 |
| **Condition aggravation rate（n,%）** | | 41，24.1 | 36，21.2 | 0.517 |
| **LOS(day)** | | 12.0(9.0,15.0) | 13.0(11.0,18.0) | 0.004 |
| **Interval of Covid-19 nucleic acid from positive to negative(day)** | | 14.0(10.0,16.0) | 15.0(12.8,17.0) | 0.004 |
| **Fatality（n,%）** | | 12，7.1 | 7，4.1 | 0.238 |

Note: WBC, white blood cell; PCT, procalcitonin; IL-6, interleukin-6; PT, prothrombin time; APTT, activated partial thromboplastin time; TBIL, total bilirubin; Cr, creatinine; BUN, blood urea nitrogen; CRP, C-reactive protein; TNI, troponin I; NT-proBNP, N-terminal pro-brain natriuretic peptide; ICU, intensive care unit; LOS, length of stay;SARS-CoV-2, severe acute respiratory syndrome coronavirus 2; RT-PCR, reverse transcription-polymerase chain reaction; CT, cycle threshold.

**Supplemental Table 2 Biochemical indexes and prognosis comparison of Early Antibody Group, Late Antibody Group and Control Group**

| **Indexes** | | **Early Antibody Group（N=77)** | **Late Antibody Group(N=93)** | **Control Group(N=170)** | **P value** |
| --- | --- | --- | --- | --- | --- |
| **ICU attending rate（n,%）** | | 23，29.9 | 19,20.4 | 39，22.9 | 0.330 |
| **Condition aggravation rate（n,%）** | | 13,16.9 | 28,30.1 | 36，21.2 | 0.099 |
| **Fatality（n,%）** | | 8,10.4 | 4,4.3 | 7，4.1 | 0.113 |
| **LOS(day)** | | 10.0(8.0,14.0)^* #^ | 13.0(10.0,17.3) | 13.0(11.0,18.0) | 0.005 |
| **Interval of Covid-19 nucleic acid from positive to negative(day)** | | 13.0(9.0,15.0)^* #^ | 15.0(11.0,18.5) | 15.0(12.8,17.0) | ＜0.001 |
| **D1** | **SARS-CoV-2 RT-PCR CT values(O)** | 24.04（21.31，28.28） | 25.32（20.70，29.33） | 23.44(16.87,28.67) | 0.059 |
|  | **SARS-CoV-2 RT-PCR CT values(N)** | 23.90（21.03，29.03） | 25.17（20.73，28.60） | 24.42(19.03,28.93) | 0.641 |
|  | **WBC,*10^9/L** | 4.85（3.93，7.70） | 4.83（3.57，7.20） | 5.24(3.97,6.97) | 0.640 |
|  | **Neutrophil,*10^9/L** | 3.46（2.83，6.50） | 3.23（2.54，5.58） | 3.59(2.52,5.51) | 0.456 |
|  | **Lymphocyte,*10^9/L** | 0.68（0.41，1.04） | 0.84（0.48，1.23） | 0.85(0.54,1.37) | 0.055 |
|  | **PCT,ng/ml** | 0.200（0.090，0.670）^*^ | 0.130（0.050，0.230） | 0.104(0.059,0.220) | 0.009 |
|  | **IL-6,pg/ml** | 71.5（23.2，147.6）^*^ | 21.7（11.3，80.4） | 19.7(7.6,49.8) | 0.005 |
|  | **D-dimer,mg/l** | 1.09（0.69，2.93）^*^ | 0.79（0.44，1.63） | 0.55(0.26.1.25) | ＜0.001 |
|  | **PT,s** | 13.1（12.1，14.0）^* #^ | 12.1（11.5，12.9） | 11.8(11.3,12.8) | ＜0.001 |
|  | **APTT,s** | 30.4（27.0，33.5）^#^ | 27.6（24.7，31.1） | 29.0(27.0,32.3) | 0.042 |
|  | **TBIL,umol/l** | 10.68（8.05，15.15） | 10.53（7.46，16.16） | 10.5(7.5,15.6) | 0.970 |
|  | **Cr,umol/l** | 80.8（59.4，107.5）^* #^ | 82.0（69.5，115.6）^*^ | 71.1(56.7,88.2) | 0.008 |
|  | **BUN,mmol/l** | 6.18（4.29，9.26） | 6.13（4.93，11.83） | 5.23(4.26,7.41) | 0.060 |
|  | **CRP,mg/l** | 39.0（11.9，118.2） | 13.5（4.4，29.6） | 11.9(3.7,39.9) | ＜0.001 |
|  | **TNI,ng/ml** | 0.02（0.01，0.08） | 0.02（0.02，0.080） | 0.02(0.01,0.03) | 0.095 |
|  | **NT-proBNP,pg/ml** | 802.1（240.9，4030.8）^*^ | 605.5（118.1，5081.1） | 279.5(64.7,1309.0) | 0.042 |
| **D7** | **SARS-CoV-2 RT-PCR CT values(O)** | 30.11（26.38，34.46） | 29.46（26.68，34.74） | 29.39（24.85，33.40） | 0.501 |
|  | **SARS-CoV-2 RT-PCR CT values(N)** | 30.95（26.03，34.18） | 28.98（26.26，32.71） | 30.29（25.24，34.30） | 0.565 |
|  | **WBC, *10^9/L** | 4.56（2.99，7.16） | 4.92（3.57，7.23） | 5.69(4.04,7.98) | 0.033 |
|  | **Neutrophil, *10^9/L** | 3.33（1.99，6.85） | 3.46（2.17，5.72） | 3.84(2.44,6.58) | 0.337 |
|  | **Lymphocyte, *10^9/L** | 0.74（0.53，1.1） | 0.75（0.50，1.09）^*^ | 1.02(0.53,1.49) | 0.015 |
|  | **PCT, ng/ml** | 0.280（0.120，0.890） | 0.360（0.100，1.540） | 0.120(0.061,0.480) | 0.060 |
|  | **IL-6, pg/ml** | 42.6（13.1，103.2） | 80.6（34.1，258.6）^*^ | 21.8(8.8,61.3) | 0.014 |
|  | **D-dimer, mg/l** | 1.65（0.55，6.62）^*^ | 1.06（0.44，1.89） | 0.77(0.53,1.93) | 0.033 |
|  | **PT, s** | 12.9（11.9，13.7） | 12.6（11.7，13.8） | 12.2(11.5,13.1) | 0.155 |
|  | **APTT, s** | 30.0（27.5，34.4） | 31.5（29.2，34.8） | 29.2(25.8,33.5) | 0.067 |
|  | **TBIL, umol/l** | 10.14（6.98，17.62） | 10.79（7.49，14.51） | 11.35(8.74,15.95) | 0.580 |
|  | **Cr, umol/l** | 73.1（58.2，105.6） | 76.3（60.5，142.5）^*^ | 65.4(51.5,86.0) | 0.013 |
|  | **BUN, mmol/l** | 8.35（4.06，13.74） | 6.58（4.29，13.26） | 5.57(3.89.8.89) | 0.044 |
|  | **CRP, mg/l** | 34.4（13.8，84.8） | 62.7（14.9，133.2）^*^ | 27.8(6.0,66.2) | 0.018 |
|  | **TNI, ng/ml** | 0.03（0.02，0.08） | 0.03（0.01，0.06） | 0.03(0.01,0.15) | 0.580 |
|  | **NT-proBNP, pg/ml** | 2737.6（634.4，11175.9）^*^ | 1959.8（354.8，3374.7） | 547.5(203.3,2222.1) | 0.020 |
| **D14** | **WBC, *10^9/L** | 5.57（3.93，7.92） | 6.14（4.78，8.44） | 6.03（4.42，8.32） | 0.469 |
|  | **Neutrophil, *10^9/L** | 4.02（2.80，6.07） | 4.65（3.19，7.58） | 3.80（2.78，5.68） | 0.331 |
|  | **Lymphocyte, *10^9/L** | 1.01（0.65，1.19） | 0.80（0.41，1.24） | 1.02（0.55，1.44） | 0.327 |
|  | **PCT, ng/ml** | 0.160（0.090，0.530） | 0.360（0.070，1.530） | 0.095（0.059，0.396） | 0.130 |
|  | **IL-6, pg/ml** | 36.7（18.0，82.3） | 15.0(9.3,303.0) | 20.7（7.3，49.8） | 0.233 |
|  | **D-dimer, mg/l** | 1.58（0.66，4.98） | 0.90(0.49,2.69) | 1.23（0.50，4.23） | 0.366 |
|  | **PT, s** | 13.2（12.3，14.0） | 13.2(12.0,13.9) | 12.3（11.8，13.3） | 0.134 |
|  | **APTT, s** | 28.7（27.0，32.5） | 29.5(26.5,33.9) | 26.8（24.7，30.5） | 0.120 |
|  | **TBIL,umol/l** | 10.88（8.46，13.35） | 11.79(8.16,17.40) | 12.43（6.91，15.30） | 0.854 |
|  | **Cr,umol/l** | 80.6（63.7，123.9）^*^ | 66.7(56.5,126.0) | 62.5（45.8，78.4） | 0.003 |
|  | **BUN, mmol/l** | 8.19（4.50，13.8） | 8.69(4.27,13.23) | 6.18（4.21，9.06） | 0.248 |
|  | **CRP, mg/l** | 21.2（7.7，61.2） | 55.9(14.0,105.7) ^*^ | 22.6（2.7，62.5） | 0.039 |
|  | **TNI, ng/ml** | 0.03（0.01，0.05） | 0.02(0.01,0.06) | 0.03（0.01，0.05） | 0.710 |
|  | **NT-proBNP, pg/ml** | 2294.0(487.8,8886.8) | 1341.7(809.5,3898.2) | 562.4（277.1，1649.9） | 0.102 |

Note: “*” represents comparing to Control Group, p<0.05; “#” represents comparing to Late Antibody Group, p<0.05. Although the p value of WBC and BUN on Day 7 of three groups’ comparison is less than 0.05. Undergoing pairwise test, the p values are more than 0.05.

WBC, white blood cell; PCT, procalcitonin; IL-6, interleukin-6; PT, prothrombin time; APTT, activated partial thromboplastin time; TBIL, total bilirubin; Cr, creatinine; BUN, blood urea nitrogen; CRP, C-reactive protein; TNI, troponin I; NT-proBNP, N-terminal pro-brain natriuretic peptide; ICU, intensive care unit; LOS, length of stay;SARS-CoV-2, severe acute respiratory syndrome coronavirus 2; RT-PCR, reverse transcription-polymerase chain reaction; CT, cycle threshold.

**Supplemental Table 3**  **Biochemical indexes and prognosis comparison of Mild Antibody Group and Mild Control Group**

| **Indexes** | | **Mild Antibody Group(N=99)** | **Mild Control Group(N=109)** | **P value** |
| --- | --- | --- | --- | --- |
| **LOS(day)** | | 12.0（9.0，16.0） | 13.0(11.0,18.3) | 0.018 |
| **Interval of Covid-19 nucleic acid from positive to negative(day)** | | 13.0（10.0，18.0） | 14.5(12.0,19.0) | 0.033 |
| **D1** | **SARS-CoV-2 RT-PCR CT values(O)** | 25.73（22.16，29.32） | 23.25(17.83,28.66) | 0.005 |
|  | **SARS-CoV-2 RT-PCR CT values(N)** | 25.35（23.03，28.75） | 24.13(19.09,28.43) | 0.062 |
|  | **WBC,*10^9/L** | 4.80（3.85，7.47） | 4.99(3.96,6.58) | 0.912 |
|  | **Neutrophil,*10^9/L** | 3.36（2.57，5.67） | 3.47(1.92,4.78) | 0.478 |
|  | **Lymphocyte,*10^9/L** | 0.85（0.47，1.26） | 1.17(0.65,1.52) | 0.018 |
|  | **PCT,ng/ml** | 0.115（0.060，0.200） | 0.080(0.053,0.140) | 0.083 |
|  | **IL-6,pg/ml** | 35.5（8.7，75.6） | 11.8(5.2,27.0) | 0.174 |
|  | **D-dimer,mg/l** | 0.90（0.51，1.90） | 0.38(0.21,0.67) | ＜0.001 |
|  | **PT,s** | 12.4（11.6，13.4） | 11.7(11.2,12.5) | ＜0.001 |
|  | **APTT,s** | 26.5（23.8，30.1） | 28.9(26.9,31.8) | 0.001 |
|  | **TBIL,umol/l** | 10.21（7.59，14.48） | 10.70(7.70,15.45) | 0.436 |
|  | **Cr,umol/l** | 78.9（59.4，95.9） | 67.2(56.8,81.7) | 0.018 |
|  | **BUN,mmol/l** | 5.67（3.81，8.01） | 4.86(4.07,6.13) | 0.103 |
|  | **CRP,mg/l** | 13.2（4.0，27.3） | 7.9(3.5,19.1) | 0.131 |
|  | **TNI,ng/ml** | 0.02（0.01，0.03） | 0.01(0.01,0.03) | 0.6 |
|  | **NT-proBNP,pg/ml** | 354.0（138.4，748.1） | 241.4(100.2,480.1) | 0.118 |
| **D7** | **SARS-CoV-2 RT-PCR CT values(O)** | 30.97（26.73，34.95） | 30.09(26.54,33.22) | 0.194 |
|  | **SARS-CoV-2 RT-PCR CT values(N)** | 30.05（26.41，34.15） | 30.81(26.34,34.33) | 0.941 |
|  | **WBC,*10^9/L** | 4.20（3.07，6.07） | 4.82(3.60,6.35) | 0.265 |
|  | **Neutrophil,*10^9/L** | 2.88（1.93，4.94） | 3.07(2.20,4.42) | 0.744 |
|  | **Lymphocyte,*10^9/L** | 0.87（0.58，1.25） | 1.04(0.54,1.65) | 0.169 |
|  | **PCT,ng/ml** | 0.120（0.060，0.510） | 0.097(0.050,0.326) | 0.150 |
|  | **IL-6,pg/ml** | 163.8（9.8，477.7） | 21.8(8.8,94.5) | 0.257 |
|  | **D-dimer,mg/l** | 0.84（0.39，1.73） | 0.60(0.35,1.39) | 0.210 |
|  | **PT,s** | 12.7（11.6，13.5） | 12.0(11.3,12.8) | 0.063 |
|  | **APTT,s** | 30.0（27.4，33.5） | 28.2(26.1,34.5) | 0.265 |
|  | **TBIL,umol/l** | 10.10（7.20，14.40） | 11.77(8.78,16.60) | 0.111 |
|  | **Cr,umol/l** | 71.8（58.5，90.1） | 59.3(50.9,74.1) | 0.004 |
|  | **BUN,mmol/l** | 5.12（3.66，9.21） | 4.45(3.43,5.71) | 0.042 |
|  | **CRP,mg/l** | 24.5（5.7，64.2） | 12.2(2.9,42.1) | 0.040 |
|  | **TNI,ng/ml** | 0.02（0.01，0.03） | 0.02(0.01,0.05) | 0.433 |
|  | **NT-proBNP,pg/ml** | 1872.7（231.0，4807.5） | 303.0(185.0,726.4) | 0.049 |
| **D14** | **WBC,*10^9/L** | 5.71（4.17，7.82） | 4.97(4.32,6.46) | 0.813 |
|  | **Neutrophil,*10^9/L** | 4.03（2.35，6.27） | 3.21(2.54,3.87) | 0.534 |
|  | **Lymphocyte,*10^9/L** | 0.84（0.57，1.31） | 1.22(0.86,1.75) | 0.077 |
|  | **PCT,ng/ml** | 0.195（0.048，0.933） | 0.067(0.050,0.427) | 0.339 |
|  | **IL-6,pg/ml** | 14.6（12.4，14.6） | 21.6(7.9,70.5) | 0.881 |
|  | **D-dimer,mg/l** | 0.71（0.50，2.78） | 0.64(0.42,1.14) | 0.411 |
|  | **PT,s** | 13.2（11.7，13.9） | 12.1(11.7,12.4) | 0.032 |
|  | **APTT,s** | 28.4（25.2，30.6） | 25.9(24.8,31.2) | 0.516 |
|  | **TBIL,umol/l** | 10.91（8.25，21.10） | 11.59(7.69,16.04) | 0.511 |
|  | **Cr,umol/l** | 66.5（54.5，108.0） | 61.2(49.0,78.3) | 0.139 |
|  | **BUN,mmol/l** | 5.17（3.39，11.91） | 4.81(3.65,6.66) | 0.455 |
|  | **CRP,mg/l** | 34.3（9.1，107.4） | 7.1(2.0,53.5) | 0.040 |
|  | **TNI,ng/ml** | 0.02（0.01，0.03） | 0.02(0.01,0.04) | 0.659 |
|  | **NT-proBNP,pg/ml** | 892.1（443.3，1746.6） | 277.1(180.2,885.9) | 0.183 |

Note: WBC, white blood cell; PCT, procalcitonin; IL-6, interleukin-6; PT, prothrombin time; APTT, activated partial thromboplastin time; TBIL, total bilirubin; Cr, creatinine; BUN, blood urea nitrogen; CRP, C-reactive protein; TNI, troponin I; NT-proBNP, N-terminal pro-brain natriuretic peptide; ICU, intensive care unit; LOS, length of stay;SARS-CoV-2, severe acute respiratory syndrome coronavirus 2; RT-PCR, reverse transcription-polymerase chain reaction; CT, cycle threshold.

**Supplemental Table 4**  **Biochemical indexes and prognosis comparison of Severe Antibody Group and Severe Control Group**

| **Indexes** | | **Severe Antibody Group(N=71)** | **Severe Control Group(N=61)** | **P value** |
| --- | --- | --- | --- | --- |
| **Fatality（n,%）** | | 9，12.7 | 7,11.5 | 0.833 |
| **D1** | **SARS-CoV-2 RT-PCR CT values(O)** | 22.37（18.71，27.03） | 24.07(16.70,29.14) | 0.771 |
|  | **SARS-CoV-2 RT-PCR CT values(N)** | 22.04（19.52，28.91） | 25.01(18.81,30.00) | 0.449 |
|  | **WBC,*10^9/L** | 5.03（3.83，7.82） | 5.75(4.09,8.08) | 0.407 |
|  | **Neutrophil,*10^9/L** | 3.46（2.72，6.78） | 4.43(2.91,7.18) | 0.413 |
|  | **Lymphocyte,*10^9/L** | 0.63（0.42，1.04） | 0.62(0.48,0.98) | 0.946 |
|  | **PCT,ng/ml** | 0.230（0.110，0.860） | 0.160(0.100,0.497) | 0.317 |
|  | **IL-6,pg/ml** | 72.1（21.8，168.4） | 38.0(14.8,71.5) | 0.104 |
|  | **D-dimer,mg/l** | 0.94（0.60，2.75） | 0.92(0.59,2.33) | 0.835 |
|  | **PT,s** | 12.6（11.8，13.9） | 12.2(11.6,13.5) | 0.298 |
|  | **APTT,s** | 31.4（28.4，34.6） | 29.3(27.2,33.6) | 0.102 |
|  | **TBIL,umol/l** | 11.09（8.56，16.87） | 9.73(6.90,20.60) | 0.538 |
|  | **Cr,umol/l** | 85.3（70.9，123.5） | 81.6(56.5,129.5) | 0.316 |
|  | **BUN,mmol/l** | 7.31（5.21，13.56） | 7.14(4.62,11.80) | 0.715 |
|  | **CRP,mg/l** | 43.6（14.2，139.8） | 52.3(10.9,94.0) | 0.160 |
|  | **TNI,ng/ml** | 0.06（0.02，0.18） | 0.03(0.01,0.05) | 0.081 |
|  | **NT-proBNP,pg/ml** | 1018.4（186.4，6459.3） | 877.5(270.0,2475.0) | 0.481 |
| **D7** | **SARS-CoV-2 RT-PCR CT values(O)** | 29.18（26.07，33.49） | 27.62(23.42,34.14) | 0.557 |
|  | **SARS-CoV-2 RT-PCR CT values(N)** | 29.77（25.12，32.62） | 27.83(24.40,33.78) | 0.492 |
|  | **WBC,*10^9/L** | 5.16（3.66，8.62） | 7.45(5.07,10.89) | 0.054 |
|  | **Neutrophil,*10^9/L** | 4.17（2.61，6.83） | 5.87(3.38,8.98) | 0.008 |
|  | **Lymphocyte,*10^9/L** | 0.64（0.39，0.86） | 0.90(0.53,1.26) | 0.002 |
|  | **PCT,ng/ml** | 0.370（0.170，1.720） | 0.157(0.078,0.717) | 0.038 |
|  | **IL-6,pg/ml** | 51.5（22.8，103.2） | 21.7(7.5,61.3) | 0.025 |
|  | **D-dimer,mg/l** | 1.66（0.79，4，71） | 1.05(0.48,3.49) | 0.197 |
|  | **PT,s** | 12.8（12.0，14.2） | 12.4(11.6,14.1) | 0.384 |
|  | **APTT,s** | 31.8（28.9，41.6） | 30.3(25.5,33.5) | 0.023 |
|  | **TBIL,umol/l** | 10.84（8.10，17.36） | 10.61(8.63,13.40) | 0.689 |
|  | **Cr,umol/l** | 92.8（60.4，200.8） | 81.7(53.7,135.2) | 0.204 |
|  | **BUN,mmol/l** | 10.45（7.18，18.25） | 7.68(5.85,11.27) | 0.098 |
|  | **CRP,mg/l** | 77.5（37.5，119.9） | 46.2(16.1,150.5) | 0.221 |
|  | **TNI,ng/ml** | 0.05（0.02，0.14） | 0.12(0.01,3.73) | 0.546 |
|  | **NT-proBNP,pg/ml** | 2268.4（664.1，4815.3） | 708.0(291.6,3210.0) | 0.091 |
| **D14** | **WBC,*10^9/L** | 6.74（4.86，11.41） | 6.60(4.70,8.87) | 0.568 |
|  | **Neutrophil,*10^9/L** | 5.37（3.53，8.01） | 5.40(3.50,7.67) | 0.537 |
|  | **Lymphocyte,*10^9/L** | 0.66（0.37，1.11） | 0.78(0.47,1.10) | 0.546 |
|  | **PCT,ng/ml** | 0.240（0.100，1.360） | 0.098(0.063,0.958) | 0.132 |
|  | **IL-6,pg/ml** | 30.9（12.8，142.3） | 19.9(7.2,42.1) | 0.116 |
|  | **D-dimer,mg/l** | 1.70（0.66，4.98） | 2.78(0.82,7.09) | 0.569 |
|  | **PT,s** | 13.4（12.6，14.3） | 13.2(11.9,15.4) | 0.620 |
|  | **APTT,s** | 30.3（28.0，33.1） | 28.3(25.0,30.8) | 0.080 |
|  | **TBIL,umol/l** | 11.50（8.80，15.10） | 12.50(6.68,15.06) | 0.878 |
|  | **Cr,umol/l** | 97.9（64.2，143.7） | 63.6(44.7,77.9) | 0.011 |
|  | **BUN,mmol/l** | 11.59（5.98，22.64） | 7.58(5.41,11.68) | 0.071 |
|  | **CRP,mg/l** | 60.9（17.6，107.3） | 38.7(10.3,95.5) | 0.184 |
|  | **TNI,ng/ml** | 0.04（0.01，0.06） | 0.05(0.02,0.08) | 0.550 |
|  | **NT-proBNP,pg/ml** | 2369.0（850.7，6805.0） | 869.6(355.0,3213.3) | 0.030 |

Note: WBC, white blood cell; PCT, procalcitonin; IL-6, interleukin-6; PT, prothrombin time; APTT, activated partial thromboplastin time; TBIL, total bilirubin; Cr, creatinine; BUN, blood urea nitrogen; CRP, C-reactive protein; TNI, troponin I; NT-proBNP, N-terminal pro-brain natriuretic peptide; ICU, intensive care unit; LOS, length of stay;SARS-CoV-2, severe acute respiratory syndrome coronavirus 2; RT-PCR, reverse transcription-polymerase chain reaction; CT, cycle threshold.
